# Upper Extremity Functional Status in Patients with Chronic Tetraplegia in Universiti Malaya Medical Centre

**DOI:** 10.21315/mjms2023.30.6.9

**Published:** 2023-12-19

**Authors:** Punithamalar Sundrasegaran, Julia Patrick Engkasan

**Affiliations:** Department of Rehabilitation Medicine, Universiti Malaya, Kuala Lumpur, Malaysia

**Keywords:** spinal injury, tetraplegia, upper extremity paresis

## Abstract

**Background:**

Persons with tetraplegia rank improved upper extremity (UE) function as the most important rehabilitation outcome because it allows them greater independence in activities of daily living (ADL). The aim of this study was to describe UE status in patients with tetraplegia using the International Spinal Cord Injury Upper Extremity Basic Data Set version 1.1 (ISCI-UE 1.1) and to determine differences in UE status between tetraplegic individuals with traumatic and non-traumatic SCIs.

**Methods:**

This cross-sectional study was conducted among patients with tetraplegia who attended the SCI rehabilitation clinic of a tertiary hospital from September 2021 to August 2022. Both upper limbs were assessed using ISCI-UE 1.1.

**Results:**

One hundred patients were included in this study, of whom 80 were men. The mean (SD) age of the patients was 54.30 (16.95) years old. In these patients, most SCIs (62%) were of traumatic origin. Two hundred UEs were evaluated, of which 109 showed good hand function (level 5) and 10 had the poorest hand function (level 1). Meanwhile, 130 UEs showed good shoulder function (level D) and 10 had the poorest shoulder function (level A). A statistically significant association with UE status (reach-and-grasp ability and shoulder function) was found in both the non-traumatic and traumatic SCI groups, with better hand and shoulder functions in the non-traumatic SCI group (right-hand, *P* = 0.004 and left hand, *P* = 0.001; right shoulder, *P* < 0.001 and left shoulder, *P* = 0.002).

**Conclusion:**

ISCI-UE 1.1 is a feasible tool for documenting UE function in patients with tetraplegia. Compared with the individuals with traumatic SCI in this study, those with non-traumatic SCI demonstrated better upper extremity functionality.

## Introduction

The prevalence of spinal cord injury (SCI) has been increasing globally and studies have shown that cervical SCI is the most common (1). Although respiratory disorders account for the leading cause of rehospitalisation and mortality among persons with tetraplegia (2–5), research has shown that these patients prioritise improved upper extremity (UE) function, as it permits them greater independence in activities of daily living (ADL) (6–8). The neurological level and completeness of the injury or lesion determine the levels of function and independence of persons with cervical SCI. The International Standards for Neurological Classification of Spinal Cord Injury (ISNCSCI) is the most frequently used tool for determining the neurological level and completeness of a lesion or injury. However, the ISNCSCI UE components do not provide information on how individuals with tetraplegia use their hands, forearms and proximal arms in complex movements (9). In these individuals, the assessment of UE function in greater detail is essential because significant improvement in UE function is required for mobility and completing basic ADL (10).

Previous research has used numerous outcome measures to classify UE function in persons with tetraplegia. However, no consensus has been reached on the most appropriate data to document the UE functions of individuals with SCI (9). The tools used to assess UE function in this population include the grasp and release test; UE function test, instrumented workstation; Sollerman hand function test; Jebsen hand function test; Minnesota manual dexterity test; action research arm test (ARAT); graded redefined assessment of strength, sensibility and prehension; and the Toronto Rehabilitation Institute-hand function test (11). As their names suggest, not all tests assess both hand and arm functions in one comprehensive test. The grasp and release, and Sollerman hand function tests are not feasible for patients with tetraplegia. Meanwhile, other tests such as the Jebsen hand function test, Minnesota manual dexterity test, and ARAT require good truncal balance, which is limited in patients with cervical SCI. Thus, their results may not reflect the actual UE function in patients with tetraplegia.

In 2014, International Spinal Cord Injury Upper Extremity Basic Data Set version 1.1 (ISCI-UE 1.1) was developed by the International Spinal Cord Society (ISCOS) to standardise the collection and reporting of data on basic findings regarding UE function in the SCI population (9). ISCI-UE 1.1 includes scores on reach-and-grasp ability, shoulder function classification, use of assistive devices, UE complications (pain, spasms, contractures and oedema), and any previous UE reconstructive surgery. Existing studies on UE function status in persons with tetraplegia are limited, especially in Malaysia (12). Therefore, the purpose of this study was to describe the UE status (function, use of assistive devices, presence of complications and reconstructive surgery) in patients with tetraplegia by using ISCI-UE 1.1 and to compare UE status between traumatic and non-traumatic SCIs.

## Methods

This was a single-centre cross-sectional study conducted among eligible patients who attended the SCI Rehabilitation Clinic of the Universiti Malaya Medical Centre (UMMC) from September 2021 to August 2022. Data were collected using ISCI-UE 1.1 by a single investigator who had undergone training with the six training cases provided by the ISCOS prior to data collection.

**Figure 1:**
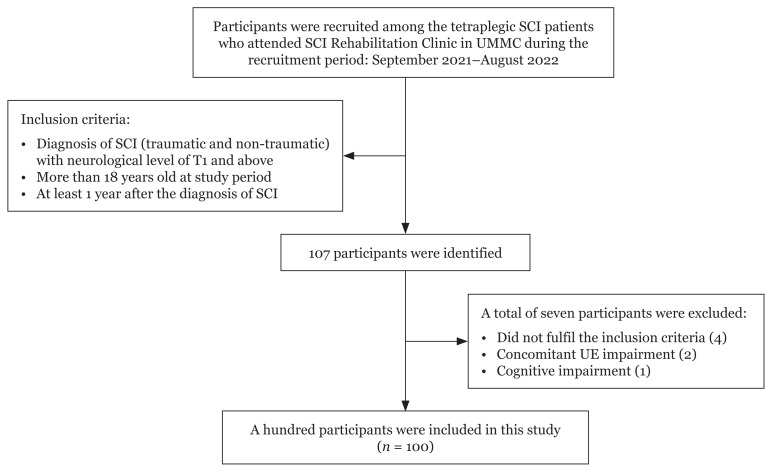
Study flowchart

All patients who attended the SCI rehabilitation clinic were screened during the study period. The inclusion criteria were as follows: age older than 18 years old at the time of the study, having SCI for at least 1 year, having traumatic or non-traumatic SCI, having complete or incomplete injury and neurological level of injury of T1 or higher. We excluded seven patients, of whom four did not meet the inclusion criteria, two had concomitant UE impairment (brachial plexus injury and stroke) and one had cognitive impairment (Figure 1). The patients’ personal and medical information, which included the registration number, age, sex, date of diagnosis, diagnosis (including the completeness of the injury), American Spinal Injury Association Impairment Scale (AIS) score and the aetiology of SCI, were collected for analysis.

The patients’ UE functions were evaluated using ISCI-UE 1.1, which is available from the ISCOS website (www.iscos.org.uk). ISCI-UE 1.1 consists of five variables: i) reach-and-grasp ability, ii) shoulder function classification, iii) use of assistive devices, iv) SCI-related complications affecting UE function and v) previous UE/hand reconstructive surgery. Reach-and-grasp ability is categorised into five levels (1–5), while shoulder function is categorised into four levels (A–D). A higher score depicts better functions. Both variables were assessed and documented separately for the right and left UEs. The scores for reach-and-grasp ability (numerical values from 1 to 5) were coupled with scores for shoulder function classification (letter values from A to D) to define the entire UE function (9). The third variable, the use of assistive devices, was comprised of devices used to enhance UE function in SCI populations and four alternative responses. The fourth variable, complications affecting UE function (including pain, spasm, contractures and oedema), was categorised as minimal, moderate or extensive. Finally, the fifth variable, previous UE/hand reconstructive surgery, was categorised as ‘yes’, ‘no’ or ‘unknown’. Those who responded ‘yes’ were asked to identify the procedure from a list of possible reconstructive surgeries for the UEs in SCI populations provided by the ISCOS.

In total, 100 patients (200 UEs) were included in this study. All statistical analyses were performed using the Statistical Package for the Social Sciences (SPSS) version 25.0 (Armonk, New York: IBM Corp.). The level of UE function was evaluated in patients with traumatic and non-traumatic tetraplegia. Other variables such as demographics and injury aetiology were presented using descriptive statistics. Continuous data were described as mean, standard deviation, median and interquartile range (IQR). Categorical data were described with frequency and percentage. The median and IQR of age between the aetiologies were determined using the Mann-Whitney U test. A chi-square analysis was used to determine the differences between traumatic and nontraumatic SCIs. The AIS scores, completeness of injury and UE functions were compared between the traumatic and non-traumatic groups using the Fisher’s exact test. A probability value of < 0.05 was used as the limit for statistical significance. All tests were two-sided.

## Results

### Demographic and Disease Specific

One hundred patients were included in this study. Their median (IQR) age at the time of this study was 55.50 years old (43 years old–68 years old), with older patients found in the non-traumatic SCI group. The youngest and oldest patients were 18 years old and 83 years old of age, respectively. A significant difference in age was found between the traumatic and non-traumatic groups (*P* = 0.028). The SCI duration ranged from 1 year to 40 years in all patients, with a median (IQR) time since injury of 8.5 years (4 years–17 years). Eighty percent of the patients were male and 20% were female. A statistically significant association was found between gender and SCI aetiology (Pearson’s chi-square test; *P* = 0.023). Sixty-two percent and 38% of the patients had traumatic and non-traumatic SCI, respectively. The distribution of the completeness of injury according to AIS classification was as follows: A = 31%; B = 11%; C = 10%; D = 49% and E = 0%. Twenty-five percent of the patients had C4 and C5 neurological levels, and 69% had incomplete injuries. Thirty-one patients had a tertiary level of education, 30 had a secondary level of education, 3 had a primary level of education, 4 had no formal education and 32 did not report their education levels. The patients’ demographic and SCI-specific characteristics are shown in Table 1.

### Upper Extremity Status

The patients’ UE functions are described in Table 2. Among the 200 UEs assessed, 109 showed good hand function (level 5), whereas 10 showed the poorest hand function (level 1). Meanwhile, 130 UEs showed good shoulder function (level D), whereas 10 had the poorest shoulder function (level A). Most patients (94%) reported that they had never used any assistive devices and only 2% of the patients had undergone a previous UE reconstructive surgery. Regarding UE complications, 65% and 9% of the patients reported minimal and extensive UE complications, respectively.

Of the two patients who had a previous surgery, one underwent fractional release of the flexor carpi radialis and pollicis longus tendon, K-wire insertion of the first metacarpal bone, tenodesis of the extensor pollicis brevis and extensor carpi radialis brevis tendon transfer. The other patient underwent surgery for the flexor pollicis longus to be looped over to the extensor pollicis longus, extensor digitorum communis tenodesis of the extensor retinaculum, transfer of the extensor carpi radialis longus to the flexor digitorum superficialis in a reversed cascade position and transfer of the brachioradialis to the flexor pollicis longus.

### Variable 1: Reach-and-Grasp Ability

The hand function score of the right UE was 5 (active extrinsic-intrinsic hand function) in 54% of the UEs, 4 (active extrinsic hand function) in 12%, 3 (active tenodesis hand function) in 12%, 2 (passive tenodesis hand function) in 17%, and 1 (no UE function at or below the elbow) in 5%. For the left UEs, the distribution of the scores was as follows: 5, 55%; 4, 12%; 3, 13%; 2, 15%; and 1, 5%.

### Variable 2: Shoulder Function

The shoulder function score of the right UE was D (good) in 66% of the UEs assessed, C in 20%, B in 9% and A in 5%. Similarly, the left UE shoulder function score was D in 64% of the UEs, C in 22%, B in 9% and A in 5%.

### Variable 3: Use of Assistive Devices

Of the patients, 94% did not use any assistive devices to perform their daily activities in our setting and only 4% used assistive devices daily, of whom 2% used them one or more times weekly.

### Variable 4: Complications Affecting UE Function

Despite the low frequency of use of assistive devices, only 9% of the patients reported having extensive complications that prevented them from performing their daily activities. Sixty-five percent of these patients complained of minimal complications and 26% had moderate complications involving their UEs.

### Variable 5: UE/Hand Reconstructive Surgery

Among the 100 patients included in this study, only 2% had undergone a previous reconstructive surgery to enhance UE function.

### Upper Extremity Function Comparison between the Traumatic and Non- Traumatic SCI Groups

**Figure 2:**
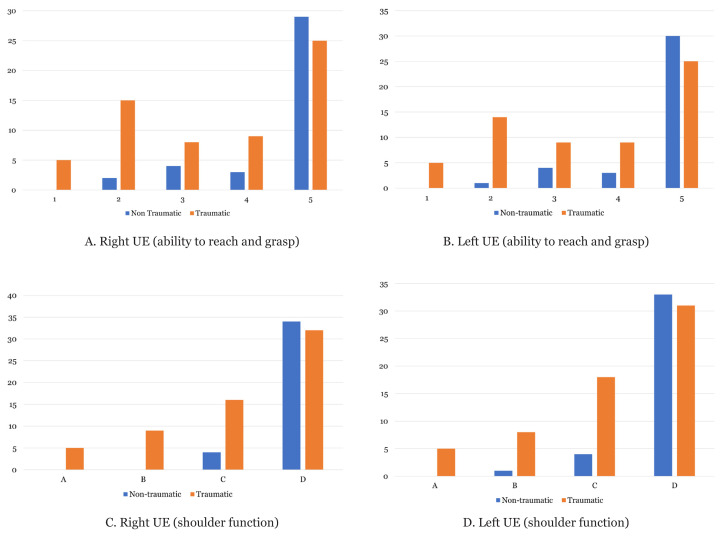
A and B show better UE (ability to reach and grasp) on the right and left UE among the non-traumatic SCI group. C and D show better UE (shoulder function) on the right and left UE among the non-traumatic SCI group

In this study, the patients with non-traumatic SCI generally had good UE function in terms of reach-and-grasp ability and shoulder function (Figure 2). Of the patients with non-traumatic SCI, 76.3% and 78.9% had a reach-and-grasp ability score of 5 for the right and left UEs, respectively. Meanwhile, 89.5% and 86.8% of the patients in this group also had good shoulder function of D on the right and left UEs, respectively. None of the patients in the non-traumatic SCI group had a reach-and-grasp ability score of 1 and a shoulder function score of A.

The patients in the traumatic SCI group had more heterogeneous findings in terms of UE function. For reach-and-grasp ability on the right UE, 8.1%, 24.2%, 12.9%, 14.5% and 40.3% of the patients had scores of 1, 2, 3, 4 and 5, respectively. For reach-and-grasp ability on the left UE, the traumatic SCI group had similar percentages for scores 1, 4 and 5. Among the patients in the traumatic SCI group, the distribution of the shoulder function scores of A, B, C and D were as follows, respectively: 8.1%, 14.5%, 25.8%, and 51.6% on the right UE, and 8.1%, 12.9%, 29% and 50% on the left UE.

The statistical significance of the comparison of UE function between the nontraumatic and traumatic SCI groups was determined using the Fisher’s exact test. Statistically significant differences in UE functional status (reach-and-grasp ability; right UE, *P* = 0.004 and left UE, *P* = 0.001) and shoulder function (right UE, *P* < 0.001 and left UE, *P* = 0.002) were found between the nontraumatic and traumatic SCI groups. Thus, the patients with non-traumatic SCI had better UE function.

A statistically significant association with completeness of injury was found in both the non-traumatic and traumatic SCI groups (*P* < 0.001) but complete injuries were more commonly found in the traumatic SCI group. No significant associations with SCI level, education level and SCI duration were found in either the non-traumatic or traumatic group. A detailed comparison of UE function between the traumatic and non-traumatic SCI groups is presented in Table 3.

## Discussion

To our knowledge, this is the first study to describe in detail the UE functions of patients with SCIs using a standardised tool (ISCI-UE 1.1). The study results indicate that the patients with non-traumatic SCI tetraplegia had better UE function than those with traumatic SCI. Owing to the heterogeneity of the non-traumatic SCI patient population, studies on non-traumatic SCI are lacking. However, in this population, the mechanisms, treatment and functional outcomes of SCI are affected by older age (13). While the epidemiology of SCI is shifting towards non-traumatic cases, research studies that focused on this group tended to have a limited number of patients or to only assess functional outcomes (e.g. functional independent measure scores and spinal cord independence measure) or use different UE assessments (11, 13, 14). Previous studies have used part of the ISCI-UE dataset (specifically ISCI-Hand) and ISCI-UE 1.0 as outcome measures to evaluate the validity and reliability of their results but have not compared UE status between non-traumatic and traumatic SCI patient groups (14, 15).

The better UE function in the non-traumatic SCI group can be explained by the incompleteness of the patients’ injuries/lesions. The patients with a traumatic aetiology had complete injuries, whereas those with a non-traumatic aetiology had incomplete injuries. Standard clinical practice promotes the use of assistive devices, but our study shows that most patients did not utilise them.

In this study, we also found that ISCI-UE 1.1 can be used during routine clinical consultations. It does not require additional clinical examinations when used for documenting UE status in patients with tetraplegia. In this study, we spent approximately 10 min completing the assessment. As this instrument was developed to ensure standardised documentation of UE status in persons with tetraplegia, we highly recommend the use of ISCI-UE in routine clinical practice.

In this study, the ISCI-UE 1.1 data set provided complementary information regarding UE function compared with the traditional ISNCSCI examination of key muscles. We found a significant difference in UE function between the SCI patients with the same AIS level (Table 4). This shows that knowledge of ISNCSCI UE key muscle strength does not reveal how individuals use the hand, forearm and proximal arm in complex movements (9). The ability to perform the described hand functions is dependent not only on the innervation per se but also on the ability to release movements against potential antagonistic muscles or changes in the fibro-elastic tissues (e.g. increased muscle tone and contractions) that counteract movements (9, 16). The combination of these hand function assessments with ADL measures would help distinguish between changes in voluntary control of muscles (e.g. changes in neurological level or myotomes) and changes in skill levels (e.g. the effects of training or non-use) (9, 17). Thus, additional knowledge from ISCI-UE 1.1 can facilitate further comprehensive rehabilitation therapy, especially for EU function in patients with tetraplegia.

One major limitation of this study is its cross-sectional design. Owing to this limitation, the usefulness of ISCI-UE 1.1 in documenting the changes in UE status over time after injury and rehabilitation could not be demonstrated. Future research should consider using this instrument to document improvement of UE status in patients who are undergoing active rehabilitation following a SCI.

## Conclusion

We advocate the use of this ISCI-UE 1.1 in daily clinical practice. When used with the ISNCSCI examination, it provides additional information regarding UE function in patients with tetraplegia, especially those with the same AIS level. Moreover, it is easy to administer because it takes less than 15 min to complete, does not have any requirements and is free or not copyrighted. ISCI-UE version 1.0 has strong inter-rater reliability and can be used as a universal language to document and monitor UE function in patients with tetraplegia.

## Figures and Tables

**Table 1:** Demographic and disease-specific characteristics

Variable	Number (%)	Median (IQR)	Range
Age (years old)		55.50 (25.00)	18.00–83.00
Gender			
Male	80 (80.0)		
Female	20 (20.0)		
Level of SCI			
T1	6 (6.0)		
C8	11 (11.0)		
C7	8 (8.0)		
C6	15 (15.0)		
C5	25 (25.0)		
C4	25 (25.0)		
C3	5 (5.0)		
C2	4 (4.0)		
C1	1 (1.0)		
AIS			
E	0 (0.00)		
D	49 (49.0)		
C	10 (10.0)		
B	10 (10.0)		
A	31 (31.0)		
Completeness			
Incomplete	69 (69.0)		
Complete	31 (31.0)		
Etiology of SCI			
Traumatic	62 (62.0)		
Non-traumatic	38 (38.0)		
Duration of SCI (years)			
1–10	56	8.5 (13 years)	1–40 (years)
11–20	26		
21–30	11		
31–40	7		
Education level			
Tertiary	31		
Secondary	30		
Primary	3		
No formal education	4		
Missing data	32		

Note: % = percentage; IQR = interquartile range; SCI = spinal cord injury; AIS = American Spinal Injury Association impairment scale

**Table 2:** Description of UE function over the right and left UE

Variable	Number (%)
Right UE (ability to reach and grasp)	
5	54 (54.0)
4	12 (12.0)
3	12 (12.0)
2	17 (17.0)
1	5 (5.0)
Left UE (ability to reach and grasp)	
5	55 (55.0)
4	12 (12.0)
3	13 (13.0)
2	15 (15.0)
1	5 (5.0)
Right UE (shoulder function)	
D	66 (66.0)
C	20 (20.0)
B	9 (9.0)
A	5 (5.0)
Left UE (shoulder function)	
D	64 (64.0)
C	22 (22.0)
B	9 (9.0)
A	5 (5.0)
Use of assistive device	
Used daily	4 (4.0)
Not daily, but one or more times weekly	2 (2.0)
Never	94 (94.0)
Complications to UE function	
Moderate	26 (26.0)
Minimal	65 (65.0)
Extensive	9 (9.0)
UE reconstructive surgery	
No	98 (98.0)
Yes	2 (2.0)

Note: % = percentage; UE = upper extremity

**Table 3:** Comparison between traumatic SCI and non-traumatic SCI

Variable	Non-traumatic SCI	Traumatic SCI	*P*-value
Age (years old)	63.50 ± 28.25	52.00 ± 23.50	0.028 (m)
Gender			
Female	12.0 (31.6)	8.0 (12.9)	0.023 (c)
Male	26.0 (68.4)	54.0 (87.1)	
Level of SCI			
C1	1.0 (2.6)	0.0 (0.0)	0.712 (f)
C2	3.0 (7.9)	1.0 (1.6)	
C3	2.0 (5.3)	3.0 (4.8)	
C4	10.0 (26.3)	15.0 (24.2)	
C5	9.0 (23.7)	16.0 (25.8)	
C6	4.0 (10.5)	11.0 (17.7)	
C7	3.0 (7.9)	5.0 (8.1)	
C8	3.0 (7.9)	8.0 (12.9)	
T1	3.0 (7.9)	3.0 (4.8)	
AIS			
A	2.0 (5.3)	29.0 (46.8)	
B	2.0 (5.3)	8.0 (12.9)	
C	4.0 (10.5)	6.0 (9.7)	< 0.001 (f)
D	30.0 (78.9)	19.0 (30.6)	
E	0 (0.0)	0 (0.0)	
Completeness			
Complete	2.0 (5.3)	29.0 (46.8)	
Incomplete	36.0 (94.7)	33.0 (53.2)	< 0.001 (c)
Right UE (ability to reach and grasp)			
1	0	5.0 (8.1)	
2	2.0 (5.3)	15.0 (24.2)	0.004 (f)
3	4.0 (10.5)	8.0 (12.9)	
4	3.0 (7.9)	9.0 (14.5)	
5	29.0 (76.3)	25.0 (40.3)	
Left UE (ability to reach and grasp)			
1	0	5.0 (8.1)	
2	1.0 (2.6)	14.0 (22.6)	
3	4.0 (10.5)	9.0 (14.5)	0.001 (f)
4	3.0 (7.9)	9.0 (14.5)	
5	30.0 (78.9)	25.0 (40.3)	
Right UE (shoulder function)			
A	0.0 (0.0)	5.0 (8.1)	
B	0.0 (0.0)	9.0 (14.5)	
C	4.0 (10.5)	16.0 (25.8)	< 0.001 (f)
D	34.0 (89.5)	32.0 (51.6)	
Left UE (shoulder function)			
A	0.0 (0.0)	5.0 (8.1)	
B	1.0 (2.6)	8.0 (12.9)	
C	4.0 (10.5)	18.0 (29.0)	0.002 (f)
D	33.0 (86.8)	31.0 (50.0)	

Note: SCI = spinal cord injury; UE = upper extremity; (m) = Mann Whitney U test; (c) = Pearson’s chi-square test; (f) = Fisher’s exact test

**Table 4:** Additional information on UE function using ISCI-UE 1.1

Patient	NLI	AIS	Aetiology	Right UE	Left UE	Description
Patient 1	C5	A	Traumatic	4D	4D	4 = Active extrinsic hand. Voluntary control of wrist and some extrinsic hand muscles allowing for grasping with or without tenodesis enabling some active opening and closing of the hand but reduced dexterity and reduction of workspace. D = Ability to reach in all directions including lifting hand above the head reflecting at least grade 3 strength in the shoulder flexors and abductors and elbow extensors.
Patient 8	C5	A	Traumatic	2C	2C	2 = Passive tenodesis hand. Passive hand functions with neither voluntary control of extrinsic and intrinsic hand muscles nor ability to actively extend the wrist. Opening and closing of the hand is only possible by supination or pronation of the forearm (passive tenodesis effect) with no active grasping movements of hand. Bimanual grasping by stabilising objects between two hands or passive tenodesis grasp is effective only in a limited workspace. C = Limited but able to reach mouth/head, with difficulty or altered movements, e.g. weak or absent pronation-supination or wrist flexion-extension.
Patient 57	C5	A	Traumatic	3C	3C	3 = Active tenodesis hand. No voluntary control of extrinsic and intrinsic hand muscles but active wrist extension allowing for passive movements of fingers dependent on a tenodesis effect. Limited single-handed grasping function in a restricted workspace. C = Limited but able to reach mouth/head, with difficulty or altered movements, e.g. weak or absent pronation-supination or wrist flexion-extension.
Patient 16	C5	D	Non-traumatic	2D	2D	2 = Passive tenodesis hand. Passive hand functions with neither voluntary control of extrinsic and intrinsic hand muscles nor ability to actively extend the wrist. Opening and closing of the hand is only possible by supination or pronation of the forearm (passive tenodesis effect) with no active grasping movements of hand. Bimanual grasping by stabilising objects between two hands or passive tenodesis grasp is effective only in a limited workspace. D = Ability to reach in all directions including lifting hand above the head reflecting at least grade 3 strength in the shoulder flexors and abductors and elbow extensors
	C5	D	Non-traumatic	5D	5D	5 = Active extrinsic-intrinsic hand. Voluntary control of extrinsic and intrinsic hand muscles with full workspace and the ability to perform different grasp forms (e.g. power grip, precision grip, lateral power pinch, precision pinch) but potential limitations of muscle strength and dexterity. D = Ability to reach in all directions including lifting hand above the head reflecting at least grade 3 strength in the shoulder flexors and abductors and elbow extensors

Note: UE = upper extremity; ISCI-UE 1.1 = International Spinal Cord Injury Upper Extremity Basic Data Sets version 1.1; NLI = neurological level of injury; AIS = American Spinal Injury Association impairment scale
